# Brain-Derived Neurotrophic Factor Levels in Cord Blood from Growth Restricted Fetuses with Doppler Alteration Compared to Adequate for Gestational Age Fetuses

**DOI:** 10.3390/medicina58020178

**Published:** 2022-01-25

**Authors:** Jara Pascual-Mancho, Pilar Pintado-Recarte, Jorge C. Morales-Camino, Carlos Romero-Román, Concepción Hernández-Martin, Coral Bravo, Julia Bujan, Melchor Alvarez-Mon, Miguel A. Ortega, Juan De León-Luis

**Affiliations:** 1 Department of Public and Maternal and Child Health, School of Medicine, Complutense University of Madrid, 28040 Madrid, Spain; japasma@gmail.com (J.P.-M.); ppintadorec@yahoo.es (P.P.-R.); concepcion.hernadez@salud.madrid.org (C.H.-M.); cbravoarribas@gmail.com (C.B.); jaleon@ucm.es (J.D.L.-L.); 2 Department of Obstetrics and Gynecology, University Hospital Gregorio Marañón, 28009 Madrid, Spain; 3 Health Research Institute Gregorio Marañón, Unidad de Investigación Materno Infantile Familia Alonso (UDIMFFA), 28009 Madrid, Spain; 4 Department of Obstetrics, Prenatal Diagnosis, Miguel Servet University Hospital, 50009 Zaragoza, Spain; 5 Laboratory of Clinical Biochemistry, Albacete Hospital, 02006 Albacete, Spain; jorgcmorales@hotmail.com (J.C.M.-C.); crroman@sescam.jccm.es (C.R.-R.); 6 Ramón y Cajal Institute of Healthcare Research (IRYCIS), 28034 Madrid, Spain; mjulia.bujan@uah.es (J.B.); mademons@gmail.com (M.A.-M.); 7 Department of Medicine and Medical Specialties, Faculty of Medicine and Health Sciences, University of Alcalá, Alcalá de Henares, 28801 Madrid, Spain; 8 Immune System Diseases-Rheumatology and Internal Medicine Service, Center for Biomedical Research Network for Liver and Digestive Diseases (CIBEREHD), University Hospital Príncipe de Asturias, Alcalá de Henares, 28801 Madrid, Spain

**Keywords:** FGR, BDNF, umbilical Doppler, brain sparing, cord blood

## Abstract

*Background and Objectives*: Fetal growth restriction (FGR) is a severe obstetric disease characterized by a low fetal size entailing a set of undesired consequences. For instance, previous studies have noticed a worrisome association between FGR with an abnormal neurodevelopment. However, the precise link between FGR and neurodevelopmental alterations are not yet fully understood yet. Brain-derived neurotrophic factor (BDNF) is a critical neurotrophin strongly implicated in neurodevelopmental and other neurological processes. In addition, serum levels of BDNF appears to be an interesting indicator of pathological pregnancies, being correlated with the neonatal brain levels. Therefore, the aim of this study is to analyze the blood levels of BDNF in the cord blood from fetuses with FGR in comparison to those with weight appropriate for gestational age (AGA). *Materials and Methods*: In this study, 130 subjects were recruited: 91 in group A (AGA fetuses); 39 in group B (16 FGR fetuses with exclusively middle cerebral artery (MCA) pulsatility index (PI) < 5th percentile and 23 with umbilical artery (UA) PI > 95th percentile). Serum levels of BDNF were determined through ELISA reactions in these groups. *Results*: Our results show a significant decrease in cord blood levels of BDNF in FGR and more prominently in those with UA PI >95th percentile in comparison to AGA. FGR fetuses with exclusively decreased MCA PI below the 5th percentile also show reduced levels of BDNF than AGA, although this difference was not statistically significant. *Conclusions*: Overall, our study reports a potential pathophysiological link between reduced levels of BDNF and neurodevelopmental alterations in fetuses with FGR. However, further studies should be conducted in those FGR subjects with MCA PI < 5th percentile in order to understand the possible implications of BDNF in this group.

## 1. Introduction

Fetal growth restriction (FGR) is a severe complication in pregnancy. It monopolizes great resources of maternal fetal research, and though clearly stated in some guidelines [1], inconsistency in terminology and definition hampers interpretation and comparison of studies. Some define fetal growth as a statistical definition of fetal size below a certain centile, referring to different thresholds for diagnosis. This also adds the possibility of including normally grown fetuses as growth restricted as well as the opposite, as it is difficult to predict the growth potential of a certain fetus [2]. The relationship between FGR and abnormal neurodevelopment has been reflected in numerous studies where the prenatal influence of poor growth on motor and executive functions in children has been explored [3,4]. Antenatal surveillance of growth-restricted fetuses is based, amongst others, on Doppler assessment [1]. The progression of FGR has been previously described and undergoes several hemodynamic phases, passing through a decrease in the estimated fetal weight centile below 10, followed by decreased pulsatility index (PI) in MCA and later, an elevation of the umbilical PI until reaching the final phase that is the alteration of the ductus venosus [5]. These stages have been related to postnatal neurodevelopment [6]. A condition deserving a highlight is the fetal Doppler adaptation to growth restriction named “brain sparing”. This phenomenon of cerebral vasodilation has been interpreted as an adaptive mechanism, but more recent studies associate it with poor results in later neurodevelopment and reviews have stated poor cognitive function and lower IQ scores [7,8].

The etiology of the neurodevelopmental alterations in FGR is not completely known. It is based on abnormal feto–maternal exchange and fetal hypoxia because of a chronic decrease in umbilical flow due to placental insufficiency [9]. Oxidative stress, neurotoxicity, apoptotic degeneration and microglial-mediated neuroinflammation are the main mechanisms related to brain injury in these fetuses [10,11,12].

On account of the difficulty of accessing the human brain in vivo, studies focused on various animal models of hypoxia have attempted to identify these intermediate mechanisms, studying neuronal growth, proliferation and survival after injury [13], the different modifications depending on the brain area studied and the severity of the growth restriction [14]. Furthermore, initial investigations on non-invasive human proton magnetic resonance spectroscopy have been ignited, showing higher lactate peaks on severely growth restricted fetal brains [15] and on not so severely restricted fetuses, as in the Sanz-Cortes study where late FGR and small for gestational age fetuses showed lower N-acetylaspartate to choline ratios, attributable to either a delay in maturational processes or to neuronal injury [16].

Other in vivo studies have tried to identify those intermediate steps centered on proteins with an important role on prenatal neurodevelopment, such as reelin on fetuses with FGR [17]. These studies try to find objective and reproductive data, easy to obtain as cord blood, to set associations to prenatal conditions, such as FGR.

Although many molecules have been described as neurotrophic biomarkers, playing important roles in neurodevelopment, neurotrophins are one of the most important actors in brain development. They are involved in neuronal differentiation and synaptic plasticity, also playing a central role in neuronal survival. Brain-derived neurotrophic factor (BDNF) is one of the most studied neurotrophins and it is closely related to neuroinflammation through its role as a modulator of neuroglia [18]. In animal models of intrauterine growth restriction, it has been widely seen that BDNF is decreased especially in the hippocampus [19,20], which is the brain region with the main expression of this neurotrophin, enhancing neuronal plasticity and relating it to memory and learning [11]. Furthermore, in vivo models have shown that the lack of microglia after cerebral ischemia increases cytokine levels, findings consistent with the protective role of microglia in the removal of waste products that indirectly relates BDNF to neuroinflammation and neuroplasticity [21].

BDNF alterations in neonates have also been studied as indicators of FGR, infection, pre-eclampsia [22], hours of rupture of membranes, corticosteroid maturation and magnesium sulfate treatment [23,24]. The largest study to date, related low levels of BDNF in dry blood tests (blood spots) taken on the first day of life in newborns with intrauterine growth restriction [25]. Furthermore, there are studies that link lower levels of BDNF with neonatal periventricular hemorrhage secondary to hypoxic-ischemic lesions [26]. Likewise, as a therapeutic approach, BDNF is being studied for neuroprotection, reducing cell apoptosis in the face of external insults, promoting specific populations of neurons in both central and peripheral nervous systems as well as after hypoxic or inflammatory brain injuries [27,28,29]. On the other hand, since the origin of neonatal BDNF in cord blood is believed to be a reflection of brain levels in animal studies [30], there are publications in the medical literature that assess BDNF levels as a predictor of behavior diseases [31] and its role in major depression, autism spectrum disorders and degenerative diseases [32,33].

Hence, due to the important role of BDNF in neurodevelopment as well as the extensive published literature on the influence of prenatal variables on their levels in newborns, this neurotrophin is a candidate for the study of intermediate processes that may relate to the prenatal insult reflected as restricted intrauterine growth and impaired postnatal neurodevelopment. Therefore, this study focuses on BDNF behavior from FGR fetuses cord blood compared to fetuses with weight appropriate for gestational age (AGA).

## 2. Materials and Methods

### 2.1. Patients Selection and Fetal Growing Assessment

Pregnant patients were prospectively recruited during their visit at the Maternity Unit of the Gregorio Marañón University Hospital. These pregnancies were dated using the cranio–caudal length at their first trimester ultrasound. Cases were selected after estimating fetal weight (EFW) by ultrasound using the Hadlock 4 formula and plotting the EFW on our own population reference tables. When the EFW was below the 10th percentile, in accordance with the ISUOG FGR criteria [1], a Doppler study was performed, assessing the pulsatility index of the umbilical artery (UA PI), the PI of the middle cerebral artery (MCA PI) and the mean PI of the uterine arteries (UtA PI) according to Ciobanu [34] and Gomez [35] reference charts, respectively, with a maximum of 7 days prior to delivery. In fetuses with adequate for gestational age weight, a Doppler study was not performed. The birth weight was obtained at the delivery room for all fetuses except for 9 FGR who were weighed within the first 12 h at the neonatal intensive care unit. Birth weights were plotted to our neonatal birth weight reference. Exclusion criteria were fetus with known congenital anomalies diagnosed prenatally or immediately postnatally, including genetic conditions, clinical chorioamnionitis, use of illicit drugs or alcohol during pregnancy or poor gestational control defined as first appointment beyond first trimester or less than 4 visits to the clinic [36]. Maternal data were withdrawn from the medical records during hospital stays, such as for preeclampsia.

To carry out the BDNF nálisis according to FGR severity, the study subjects were divided in two groups. Group A contained fetuses with weight appropriate for gestational age (AGA) birth weight ≥ 10th percentile. Group B was made up of FGR fetuses (birth weight < 10th percentile) with abnormal umbilical or cerebral Doppler study. Further detailed Doppler assessment in FGR fetuses was performed in order to assess the effect of brain vasodilation on BDNF levels. For that reason, we divided group B into two subgroups: fetuses with decreased MCA PI < 5th percentile and fetuses with increased UA PI > 95th percentile in order to assess the effect of cerebral vasodilation on BDNF levels.

### 2.2. Sample Collection, Initial Processing and Storage

Samples were collected at the time of delivery, prior to placenta evacuation and deposited into standard clinical tubes containing lithium heparin. The blood was centrifuged within the first hour of birth at the Biochemistry department, by the on-call laboratory technicians. Eppenddorf aliquots, a minimum of one and a maximum of three, coded with the study identifier were stored in racks in a Thermo Scientific Fisher Forma freezer at −86 °C. Thawing was carried out at room temperature and on only one occasion, no subsequent freezing was performed.

### 2.3. Determination of BDNF in Umbilical Vein by ELISA Methodology

BDNF determination was achieved by the Quantikine Human BDNF Immunoassay assay (R&D Systems) according to the manufacturer’s instructions. This is a sandwich-type solid phase ELISA. Anti-BDNF antibodies are immobilized on the surface of the wells of the microplate. These antibodies capture the BDNF contained in the samples, controls and calibrators and after washing a second antiBDNF, peroxidase enzyme-conjugated antibody is added, leading to a colorimetric reaction by adding the substrate (3, 3′, 5, 5′-Tetramethylbenzidine), emitting a signal proportional to the BDNF concentration. The absorbance of each sample is read spectrophotometrically at a wavelength of 450 nm. Generation of a standard curve allows for identification of the protein concentration. The intra assay and inter assay coefficients of variation for the ELISA were 5 and 9, respectively. All samples were assayed in duplicate.

### 2.4. Statistical Analysys

For the statistical analysis, IBM SPSS Statistics V21.0 was used. Differences between the groups of study were assessed using chi-square or non-parametric Mann-Whitney test, based on the normal distribution of the variables in these different groups. When assessing the influence of FGR on BDNF levels a multivariable linear regression model was performed in order to adjust for fetal variables.

## 3. Results

Our sample consisted of 130 subjects: 91 in group A (AGA fetuses); 39 in group B (16 FGR fetuses with MCA PI < 5th percentile and 23 with UA PI > 95th percentile). Fetal Doppler measurement was performed a mean of 3 days before delivery.

Obstetric and neonatal characteristics of the two groups are shown in Table 1. The characteristics surrogated to severity of FGR, such as cesarean section, admission to neonatal care unit and intraventricular hemorrhage were higher in group B. Birth weight, weight centile and gestational age were higher in group A compared to group B. Other variables, such as preeclampsia and increased PI UtA, also had different frequencies. Conversely, inflammatory markers were controlled by leukocytosis and there were no significant differences observed.

The FGR group had 41% of fetuses who required corticosteroids for lung maturation and 23% neuroprotection with MgSO_4_ due to prematurity. The four cases of intraventricular hemorrhage diagnosed during admission to neonatal care were limited to the germinal matrix.

**Figure 1:**
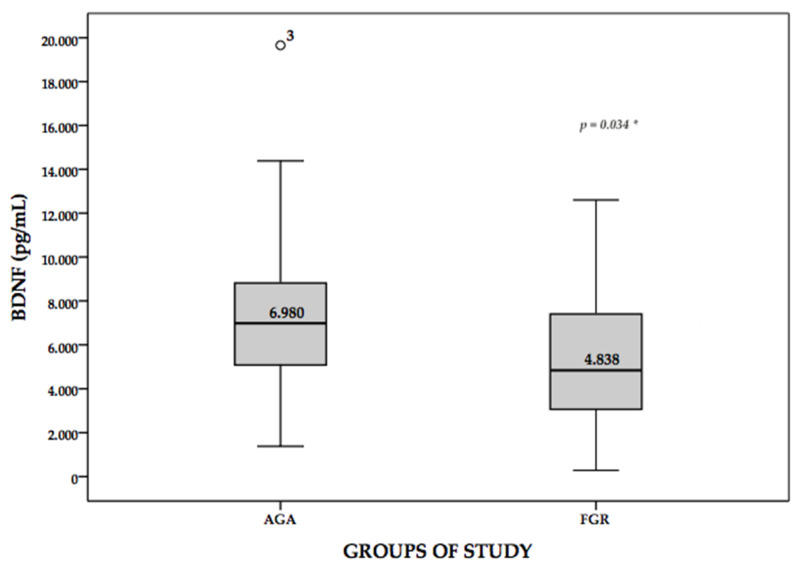
BDNF Box plot across study groups. *p* * is adjusted for gestational age with linear regression.

Figure 1 shows the BDNF values according to the groups under study. We found significant BDNF differences between medians in groups with non-parametric U Mann–Whitney test (*p* = 0.002). As fetuses with impaired growth were more likely to be preterm, we adjusted for gestational age to see the stability of the association with a linear regression (*p* = 0.034).

**Figure 2:**
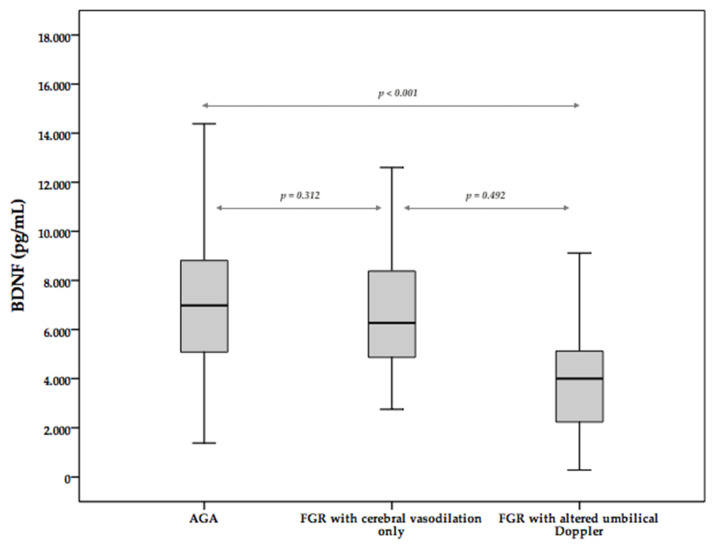
BDNF Box plot with subgroup of fetuses with brain sparing. *p* adjusted for gestational age with linear regression.

Subgroup analysis to assess the effect of decreased MCA PI on BDNF was performed. Clinical characteristics and differences between both groups are shown in Table 2. BDNF levels showed a downward trend, but no differences were found. AGA fetuses and FGR fetuses with decreased MCA PI showed similar BDNF levels. Within FGR fetuses, no differences were found between fetuses with decreased MCA PI and FGR fetuses with UA PI >95th percentile (Figure 2).

## 4. Discussion

So far, this study is the first to demonstrate a decrease in BDNF on FGR fetuses with a fetal Doppler alteration. This difference was mainly due to the low BDNF levels in the subgroup of fetuses most severely affected with an increased umbilical Doppler PI, as it is shown in Figure 2. BDNF concentration in FGR fetuses with decreased MCA PI exclusively did not differ from the other fetuses, either AGA or FGR, with UA PI > 95th percentile. Perinatal variables from FGR fetuses were different from AGA in those variables subrogated to the growth restriction environment. Preeclampsia and increased uterine artery PI are risk factors for growth restriction due to impaired placentation, and prematurity is common amongst those fetuses as the optimal time of delivery is still under discussion [37]. This explains why perinatal variables linked to gestational age, such as lung maturation and neuroprotection with MgSO_4_ as well as preeclampsia rate and pathological UtA PI linked to impaired placentation, were more likely on FGR group. Other variables also linked to either prematurity and FGR, such as intraventricular hemorrhage, cesarean section rate and neonatal care admission, were more frequent in the FGR group.

Studies in humans have also demonstrated decreased levels of BDNF in FGR fetuses [25,38,39], regardless of adaptive fetal Doppler status. However, not all studies are consistent with our findings. Malamitsi-Putchner observed there were no differences in BDNF between growth-restricted and adequate newborns [40]. These study groups had no differences in fetal Doppler; in fact, it was a recommendation that they carried out future studies. Other studies from the same group evaluated fetal BDNF behavior in diabetic mothers, seeing that the levels were lower than the healthy controls in the latter, regardless of the FGR degree they had [38]. A decreased neonatal BDNF level in diabetic mothers has been observed [41,42], and this effect could mask the one caused by the growth defect since it was diagnosed by a birth weight centile lower than 5 exclusively without Doppler evaluation. Another recent study found no differences between very low birth weight FGR and very low birth weight AGA fetuses when assessing trophic biomarkers as BDNF or vascular endothelial growth factor (VEGF). FGR criteria was again only a birth centile threshold of 10th centile [43] without any examination of Doppler status. Paradoxically, these groups had no birth weight differences. With this in mind, our study cohort had strict selection criteria, besides a birth weight centile classification for FGR condition; we also applied Doppler criteria, either umbilical or cerebral. This condition ensures a sample where healthy fetuses that are small for gestational age are not included, thereby overcoming one of the difficulties in the design of FGR studies.

Besides a rigorous selection of FGR fetuses, these studies have to deal with confounding factors. Brain injury is sometimes overcome by prematurity in severe FGR; for that reason, an adjustment for gestational age is advised. In our study, the association of the severity of growth restriction with decreased BDNF persisted after adjustment for gestational age. Another study from our group observed on healthy term newborns that BDNF cord blood levels decrease as gestational age at delivery increases (unpublished data). This fact would also confirm a low BDNF on FGR despite its lower gestational age. In order to cope with the prematurity confounding factor, Antonakopoulos et al. performed a BDNF analysis on amniotic fluid on ongoing pregnancies. They found higher levels of BDNF on amniotic fluid from small for gestational age (SGA) fetuses assessed early in the second trimester. They did not study Doppler nor fetal growth status at the time of amniocentesis as it was performed for other reasons, finding BDNF levels on large for gestational age and SGA fetuses [44].

Another factor to consider is the treatment with corticoids, as it has been related to high BDNF levels [23]. In our study, the FGR group had a higher proportion of antenatal steroids for lung maturation (41%) but despite that fact, this group had the lowest concentrations still after gestational age adjustment.

FGR fetuses have a described mechanism of adaptation to preserve important body functions called “brain sparing”. In general, it has been observed that brain vasodilation is associated with lower scores on cognitive neurodevelopmental scales at 2 years [45]; however, regarding motor alterations or low scores on early scales, no differences have been seen when comparing to newborns without brain redistribution. This probably reflects cognitive alteration rather than motor function, as attributed to the frontal cortex [7]. Regarding our fetuses with decreased MCA PI exclusively, we noted the finding of a decrease in BDNF concentration although this was not statistically significant, especially after gestational age adjustment. When the target is set to study the consequences of “brain sparing”, early and late FGR is an important factor as many of these fetuses with late onset FGR will have a slower clinical progression and might be more likely to reach term [6]. Studies with larger numbers should focus on this group of patients and long-term neurodevelopment assessment is encouraged in matched case-control studies.

However, our study has some limitations, not only in the number recruited, but also the possible confusion in the face of variables associated with the severity of FGR. Ventricular hemorrhage occurred, although it was limited to germinal matrix, and intraventricular hemorrhage can be associated with lower levels of BDNF [26]. Although we controlled for prenatal malformations, known genetic conditions at birth and infectious diseases, one of the variables that has been observed to modulate BDNF expression is maternal obesity and we lacked this data [41]. Moreover, we did not perform Doppler exam on AGA fetuses; this group might include fetuses who did not reach their growth potential, although the interquartile range was from 43 to 88. Regarding prematurity, we tried to overcome this bias through linear regression and gestational age adjustment. This handicap is present in many studies focused on FGR. Assessing brain development, neurotrophic factors and postnatal neurodevelopment with age matched controlled studies is of paramount importance but also difficult to achieve as growth restriction is linked to prematurity and healthy preterm fetuses are difficult to identify [37].

Even considering these BDNF differences in FGR, due to the fundamental role of this neurotrophin in prenatal neurodevelopment, our study lacks postnatal follow-up. Future studies of BDNF levels on growth restricted fetuses and linking to the evaluation of subsequent neurodevelopment are necessary to elucidate the intermediate mechanisms that cause such postnatal alteration. These should also serve to identify individuals at increased neurological risk and assess future intervention actions as it has already been studied in hypoxic brain injuries in postnatal Noxa [46].

## 5. Conclusions

We have observed a significant decrease in cord blood BDNF in FGR with Doppler alteration compared to AGA fetuses. This difference was greater between AGA and FGR fetuses with UA PI > 95th percentile. Decreased MCA PI in FGR fetuses needs further study as we could not find statistical differences on BDNF cord blood concentration when compared to AGA fetuses, although lower levels were observed.

## Figures and Tables

**Table 1:** Bivariate analysis of maternal and neonatal clinical characteristics of the study groups.

	GROUP A AGA *N* = 91	GROUP B FGR *N* = 39	*p*
Maternal age (years) M (IQR)	32 (7)	34 (5)	NS
Gestational age (weeks) M (IQR)	38 (4)	35 (5)	<0.001
Fetal sex (female) *n* (%)	49 (54)	15 (38)	NS
MgSO_4_ *n* (%)	0	9 (23)	<0.001
Lung maturation *n* (%)	1 (1)	16 (41)	<0.001
Cesarean section *n* (%)	11 (12)	25 (64)	<0.001
UtA PI > p95 *n* (%)	N/A	18 (46)	N/A
Preeclampsia *n* (%)	1 (1)	6 (15)	0.005
Birth weight (g) M (IQR)	3290 (650)	1750 (870)	<0.001
Weight centile M (IQR)	65 (45)	0 (1)	<0.001
pH AU M (IQR)	7.29 (0.11)	7.26 (0.09)	NS
Cord blood leukocytes (number/uL) M (IQR)	15,600 (6500)	12,700 (7500)	NS
Neonatal care admission *n* (%)	6 (6.6)	25 (64)	<0.001
Intraventricular hemorrhage *n* (%)	0	4 (10)	0.007
BDNF (pg/mL) M (IQR)	6980 (3735)	4838 (4724)	0.001

AGA: adequate for gestational age weight. FGR: fetal growth restriction; UtA PI: uterine artery pulsatility index PROM: premature rupture of membranes; IQR: interquartile range; M: median; NS: no significative. N/A: not applicable.

**Table 2:** Bivariate analysis of maternal and perinatal features between clinical subgroups.

	AGA *N* = 91	FGR with MCA PI < 5th Exclusively *N* = 16	*p*
Maternal age (years) M (SD)	32 (28–35)	34 (31–35)	NS
Gestational age (weeks) M (IQR)	38 (36–40)	36 (35–38)	0.027
Fetal sex (female) *n* (%)	49 (54)	9 (56)	NS
MgSO_4_ *n* (%)	0	3 (19)	0.003
Lung maturation *n* (%)	1 (1)	3 (19)	0.01
Cesarean section *n* (%)	11 (12)	10 (63)	<0.001
UtA PI > p95 *n* (%)	N/A	4 (25)	N/A
Preeclampsia *n* (%)	1 (1.3)	4 (25)	NS
Birth weight (g) m (SD)	3302 (442)	2069 (412)	<0.001
Weight centile M (IQR)	65 (43–88)	1 (1–3)	<0.001
pH AU M (IQR)	7.29 (0.11)	7.27 (0.08)	NS
Cord blood leukocytes (number/uL) M (IQR)	15,600 (6500)	14,700 (7225)	NS
Neonatal care admission *n* (%)	6 (6.6)	6 (37.5)	0.002
Intraventricular hemorrhage *n* (%)	0	0	NS
BDNF (pg/mL) m (SD)	6980 (3735)	6268 (3539)	NS

PROM: premature rupture of membranes; AGA: adequate for gestational age weight. IQR: interquartile range; SD: standard deviation. NS: no significative. N/A: not applicable.

## Data Availability

The data used to support the findings of the present study are available from the corresponding author upon request.
